# From behavioral synchrony to language and beyond

**DOI:** 10.3389/fnint.2024.1488977

**Published:** 2024-12-11

**Authors:** Katherine Eulau, Kathy Hirsh-Pasek

**Affiliations:** ^1^Temple Infant and Child Laboratory, Temple University, Philadelphia, PA, United States; ^2^The Brookings Institution, Washington, DC, United States

**Keywords:** behavioral synchrony, neural synchrony, joint attention, social contingency, EEG, fNIRS, language development, cognitive development

## Abstract

Decades of research on joint attention, coordinated joint engagement, and social contingency identify caregiver-child interaction in infancy as a foundation for language. These patterns of early behavioral synchrony contribute to the structure and connectivity of the brain in the temporoparietal regions typically associated with language skills. Thus, children attune to their communication partner and subsequently build cognitive skills directly relating to comprehension and production of language, literacy skills, and beyond. This has yielded marked interest in measuring this contingent, synchronous social behavior neurally. Neurological measures of early social interactions between caregiver and child have become a hotbed for research. In this paper, we review that research and suggest that these early neural couplings between adults and children lay the foundation for a broader cognitive system that includes attention, problem solving, and executive function skills. This review describes the role of behavioral synchrony in language development, asks what the relationship is between neural synchrony and language growth, and how neural synchrony may play a role in the development of a broader cognitive system founded in a socially-gated brain. We address the known neural correlates of these processes with an emphasis on work that examines the tight temporal contingency between communicative partners during these rich social interactions, with a focus on EEG and fNIRS and brief survey of MRI and MEG.

## 1 Introduction

Over the past 20 years, the scientific world of neural synchrony has exploded with thousands of publications by researchers around the globe. This burgeoning field builds on decades of behavioral science demonstrating that the timing and content of back-and-forth human to human interaction has significant relations with child outcomes (e.g., Tomasello and Todd, 1983; Tomasello and Farrar, 1986; Bakeman and Adamson, 1984; Adamson et al., 2014; Tamis-LeMonda et al., 2014; Hirsh-Pasek et al., 2015). By way of example, what Kuhl called a socially-gated brain might well hold the seeds not only for later social development like attachment, but also demonstrate how aspects of the social environment are pivotal for cognitive growth (Kuhl, 2007). In this review, we examine behavioral data linking early socially contingent interactions with language outcomes. We then move to how neural synchrony is enabling moment-to-moment analysis of the aspects of the behavioral environment that make up the mechanisms behind that language growth. In a third section, we touch on nascent ideas that posit how this early neural synchrony might go beyond social development to influence cognitive development more broadly, in attention, problem solving, and executive function skills.

## 2 Kuhl’s insight

In what has become a classic paper published in *Developmental Science* in 2007, Kuhl floats the argument that the social brain serves as a mechanism for “gating” language learning. In early childhood, she argues, infants learn “statistically,” and an infant’s social partner serves to guide the attention and arousal of the infant and to provide the correct information at the right times (Kuhl, 2007). The idea is further developed in a 2009 *Science* article from Kuhl and her team (Meltzoff et al., 2009) positing three social skills unique to human development guide learning: imitation, shared attention, and empathetic understanding. This insight begins to shape the idea that social development, as organized in the brain, might prove a foundation for the later learning of language as well as for other cognitive behaviors.

## 3 Where social contingency meets language

The field of psychology often studies social development and cognitive development in separate bins, with separate theories and methods. Discussion of social and cognitive development is often relegated to separate journals, conferences, and department wings, with language providing a link between the fields, as it marks a central feature of both cognition and social interaction in development. In the social arena, the dominant developmental theory concerned the role of attachment and family engagement. Many who studied attachment in the 1960s and 1970s, from Bowlby to Ainsworth to Sroufe (e.g., Bowlby, 1969; Ainsworth, 1964; Ainsworth, 1969; Ainsworth, 1979; Sroufe, 1979; Sroufe, 1985; Sroufe et al., 1983) held attachment as the holy grail of developmental psychology, though constrained their theory to social–emotional outcomes like relationship development (e.g., Main and Weston, 1981; Verschueren and Marcoen, 1999), emotional regulation (e.g., Main and Solomon, 1986; Raver, 1996), externalizing symptoms (e.g., Belsky, 2002), and social competence (e.g., Sroufe, 2005; Sroufe et al., 2009). A child’s attachment to his mother, they argued, was central to social development. Experimental protocols were developed to assess attachment style in children (e.g., Ainsworth, 1979) and social interaction was measured under tightly controlled conditions via eye gaze tracking (e.g., Scaife and Bruner, 1975).

Many strands converge to form a rope from social development to language development. Among them were Bakeman and Adamson’s joint engagement (Bakeman and Adamson, 1984), Tomasello’s joint intention (Tomasello et al., 1998), and Trevarthen’s intersubjectivity (Trevarthen and Aitken, 2001). Roger Bakeman and Lauren Adamson were among the first to connect early social interaction to an outcome that was not quintessentially social. In their seminal work, they argued that social input and interaction were central to language development—a point also made by researchers like Lois Bloom, Catherine Snow and Elizabeth Bates, among others (e.g., Bloom et al., 1996; Bates et al., 1975; Snow, 1977; Bretherton et al., 1979). Thus, a more holistic take on what was termed “joint attention” grew from early attempts to record social interaction via behavioral measures such as gaze following (e.g., Scaife and Bruner, 1975). Joint attention, they argued, is more than shared visual gaze, but a shared attentional state. In other words, beyond the volitional visual attention of an infant to an object, a fundamental socio-cognitive skill emerges. The infant and the communication partner share attention to an object, but *also* to one another’s socio-cognitive state (Bakeman and Adamson, 1984).

Over the course of several decades, research established that a child’s attention develops from visual attention, to shared attention, supported joint attention, coordinated joint attention, and symbol-infused coordinated joint attention (Bakeman and Adamson, 1984; Adamson et al., 2004). Coordinated joint attention, according to Bakeman, Adamson, and colleagues is a social interaction in which the child coordinates her attention with another person and the object with which that person is engaged (Bakeman and Adamson, 1984). While joint attention is not an inherently linguistic activity, symbol-infused joint engagement is the expansion of coordinated joint attention into a communicative interaction with meaningful symbols (Adamson et al., 2004). Numerous studies investigated the relation between early dyadic social interactions and language development (e.g., Tomasello and Farrar, 1986; Akhtar et al., 1991; Tamis-LeMonda et al., 1998; Hirsh-Pasek et al., 2015).

As a focal point for developmental research in both cognitive psychology and social psychology, the joint attention literature now boasts several thousand peer-reviewed publications and tens of thousands of Google Scholar hits from a broad range of media outlets covering a variety of research methodologies, sample characteristics, and analytical techniques. Over the last several decades, the rope linking social and language development has included joint intention, intersubjectivity, joint engagement, and social contingency, all broadly referring to the dyadic social interaction involving mutual attention to one’s social partner and an object. Terminologies have varied, but all have pointed to the developmental value of these social interactions as it relates to language development.

Tomasello added that joint attention is a rich social-cognitive-developmental process that includes not just visual acuity or gaze following, but symbolic gesture, vocal or verbal communication, imitation, and social referencing (Tomasello, 1988). Based upon primate observations, for example, Tomasello hypothesized that what chimpanzees lacked in social interaction was joint *intention* (Tomasello et al., 1998). Intention, he argued, is an innately human characteristic of social interactions, implying that human communication partners *share understanding* about the focus of the interaction and, of even higher order, share awareness of the interaction itself (Liszkowski et al., 2004; Carpenter et al., 1998; Tomasello et al., 2005).

Based on observations of ape and human development, Trevarthen, Tomasello, and others concluded that the social cognition sufficient for symbolic communication development is unique to human infant development (Tomasello et al., 1998; Trevarthen and Aitken, 2001). Human social development, Trevarthen and others argued, progresses from a dyadic “natural sociability” early in infancy, called primary intersubjectivity, to a more robust social cognition, secondary intersubjectivity, that moves the dyadic social interaction beyond two human social partners to include object awareness, sometimes referred to as joint attention or triadic attention (Trevarthen and Aitken, 2001; Terrace et al., 2022). Together, strong dyadic *and* triadic skills, primary and secondary intersubjectivity, found symbolic communication development (Trevarthen and Aitken, 2001; Oller et al., 2019; Terrace et al., 2022).

Aside from comparison to non-human infants, observation and experimental manipulation provide additional perspective on social development. For example, the expectations of each communication partner of the other as early as infancy are conveyed by infant behaviors such as frustration when adult communication partners abandon interactions or fail to acknowledge new bids (Dunham and Moore, 1995). In one study from Tomasello’s team, language skills increased on a weekly timescale with higher levels of joint attention concurrently *and* later into development (Tomasello and Todd, 1983). Tomasello also tested this hypothesis experimentally in toddlers; at 14-to 23-months, a toddler learned the names of novel objects better when his communication partner followed the child’s focus than when redirected (Tomasello and Farrar, 1986). Tomasello described these joint attention interactions as “hot spots” for language development. Children learn explicit language skills and both partners practice mutual attunement, improving later explicit teaching and learning (Tomasello, 1988).

Research connecting early social interactions and language outcomes prompted investigation of which features of the interaction might propel high quality interactions and language outcomes. Dunham and Dunham emphasized *following-in,* in which caregivers follow the attentional focus of the child rather than leading or directing (Dunham and Dunham, 1990; Dunham et al., 1993). Akhtar and colleagues found, for example, that children whose focus was redirected rather than followed at 13 months of age had lower caregiver-reported vocabularies at 22 months (Akhtar et al., 1991). Thus, naturalistic paradigms like those employed by Tomasello’s and Dunham and Dunham’s teams in the home and lab offered one way to investigate joint attention—in context.

Lab manipulations via tightly controlled interactions under novel conditions or with novel social partners offered another perspective on joint attention (e.g., Mundy and Gomes, 1998; Morales et al., 2000a). Such studies rely on breaking joint attention into derivative social and cognitive skills and traits that can be tested with a novel communication partner such as initiating (Mundy et al., 2000) and responding to joint attention, also known as gaze following (Morales et al., 2000a; Morales et al., 2000b), temperament (Mundy, 1995; Mundy and Gomes, 1998; Morales et al., 2000b), and initiating and responding to behavior regulation (Mundy and Gomes, 1998). Discretizing portions of the social interaction by maintaining control over the order of events, stimuli presented, and even language used allowed the researchers to collect tidy, reliable data on children’s responses to social bids during interactions.

By definition, however, joint *engagement* requires both social partners to influence one another and when measuring joint engagement, the focus is on dyadic characteristics (Adamson et al., 2014) in more ecologically valid situations. Child engagement states range from onlooking, to passive joint engagement, and finally coordinated joint engagement (Bakeman and Adamson, 1984). Thus, the highest quality interactions occur when the partners exchange awareness of one another.

Over the course of development, as the child’s cognitive and social skills grow, the caregiver is initially responsible for scaffolding or supporting these bouts of joint attention. Joint attention, in moment-to-moment caregiver-child interactions, Tamis-LeMonda argued, relies on the *contingency* of the social partners to one another (Tamis-LeMonda et al., 2014). The caregiver must respond to the child with language that is semantically related (Rowe, 2012; Rowe and Snow, 2020; Lohaus et al., 2001) and within a reasonable timeframe (Tamis-LeMonda et al., 2001; Bloom et al., 1996). These features of caregiver-child social interaction have also been investigated via experimental manipulation and clinical intervention (e.g., Landry et al., 2008; Roberts and Kaiser, 2015; Leech et al., 2018; Leech and Rowe, 2021; McGillion et al., 2017).

### 3.1 Semantic contingency

As one cornerstone of early social interactions, semantic contingency refers to the relatedness of the communication partner’s verbal productions to the child’s vocalizations, verbalizations, or direction of visual attention (Tamis-LeMonda et al., 2014). Rowe and Snow described three dimensions of semantic relatedness in caregiver utterances that predict word learning: conceptual, linguistic, and interactive input (Rowe and Snow, 2020).

#### 3.1.1 Conceptual input

Conceptual input early in development is concrete and tightly tied to present objects. Indeed, Smith and Yu argue that infants require the support of communication partners to provide clear, accurate vocabulary for relevant referents (Smith and Yu, 2008). Relevant referents are most often selected by the child via physical action or visual gaze (Pruden et al., 2006; Moore et al., 1999). Symbol-referent pairs rely on perceptual salience from the perspective of the child and awareness of the adult social partner of that which is salient to the child. Practically, this means that attuned adult social partners notice the multisensory attention of the child and provide symbols linked to that focus of attention (Suarez-Rivera et al., 2022; Suarez-Rivera et al., 2023). Children learn from repeated and consistent presentation of symbol and object pairs, a process some refer to as statistical learning (Saffran et al., 1996; Saffran, 2003; Kuhl, 2004). Infants must attend to only verbal input that relates to present stimuli, pair that symbol with the object, then repeat the process numerous times to “map” the symbol to the referent (Smith and Yu, 2008; Saffran, 2020). The perceptual salience of the referent guides the learning of the symbol and often refers to the object in the child’s hands, though can refer to the gaze of the child (Yu and Smith, 2012). Thus, rich, dyadic learning moments are derived from joint engagement around the child’s interest and multisensory direction of focus, paired with the adult social partner’s scaffolding and facilitation of symbol-referent pairs.

As infants grow into young children, semantic content from caregivers will increase in complexity to include abstract or hypothetical concepts. Additionally, research shows that children’s brains use prior experience to shape how they attend to and learn from adult models (Lew-Williams and Saffran, 2012). That is, children learn language best when they are offered clear examples of age-appropriate language that relate to their focus of attention. For infants, this will be the concrete, here and now; for toddlers and preschoolers, this may be the past, future, or hypothetical occurrence. For example, Leech and colleagues trained parents on decontextualized language modeling as a communication strategy, which subsequently improved preschoolers’ abstract, or decontextualized language, conversational turns, and vocabulary (Leech et al., 2018; Leech and Rowe, 2021). Infants and young children rely on their social partners to be clear communicators tightly attuned to their environment, increasing the odds that the language provided will match the referent of interest, whether this involves shared attention in infants or sophisticated hypothetical musings in preschoolers.

#### 3.1.2 Linguistic input

Linguistic input refers to the nature of the speech or language input itself. Early in development, this presents as infants’ preference for infant-directed speech, for example, which is characterized by prosodic fluctuations, short phrases, and limited vocabulary (e.g., Fernald and Kuhl, 1987; Golinkoff et al., 2015; Ferjan Ramírez et al., 2019). Later as toddlers and preschoolers, these features become lexical diversity, grammatical complexity, and, ultimately, conversational discourse (Rowe and Snow, 2020). Linguistic input is a measure of the quality of language the child hears and is distinct from the quantity of language (Hirsh-Pasek et al., 2015). As children grow, the use of a variety of vocabulary and grammatical structures predicts the child’s later language skills (e.g., Huttenlocher et al., 2010; Rowe, 2012; Weizman and Snow, 2001). Here, adults must balance providing sufficient repetition while also managing novelty. As their linguistic developmental environments increase in complexity, so will children’s language. Adult social partners, thus, have the opportunity to curate access to those more complex models as mastery of earlier concepts occurs.

#### 3.1.3 Interactive quality

The interactive quality of caregiver input is reflected in the carefully timed responses that follow-in on the child’s interest, vocalization, or verbalization (Rowe and Snow, 2020; Dunham and Dunham, 1990). Before even using words, infants respond more, and in kind, to turn-taking vocalizations that are phonologically contingent on the infant’s production, creating a socially reinforcing feedback loop (Goldstein and Schwade, 2010; Elmlinger et al., 2023). In other words, caregivers’ responses to infant babbling reinforce the infant behavior—the infant babbles and the caregiver responds vocally, causing the infant to babble back; and so begins a dance. The adult responds with the same or similar sounds and the infant responds. In this pattern, the infant may try new sounds and eventually new words. These are the roots of phonological and eventually vocabulary development. The contingency of the adult’s response is essential to this loop, however; if the adult’s production is too different from the infant’s or the word provided by the adult does not relate to the referent, the child may abandon the interaction (Dunham and Moore, 1995). Research shows this effect holds for vocabulary development. McGillion and colleagues, for example, provided caregiver training at 12 months on a semantic contingency similar to following-in—noticing the child’s interest and talking about it—and found that for lower socioeconomic status (SES) infants whose parents were trained, monthly vocabulary growth was significantly larger such that children of trained caregivers had, on average, a four-month advantage compared to their also lower SES peers of untrained caregivers at 18 months (McGillion et al., 2017).

### 3.2 Temporal contiguity

Interactive input, of course, would not be interactive were the timing of the turns misaligned. Central to the socio-cognitive learning environment constructed by dyadic interactions, then, is *timing*. Tamis-LeMonda describes this feature as “temporal contiguity.” In other words, the social partner’s response to the child’s attention or bid for interaction must be within an expected timeframe for the referent and the response to be paired (Tamis-LeMonda et al., 2014). As early as nine months, brain activity reflects lexical semantic processing when infants are exposed to verbal responses within 2000 ms, with the strongest brain response occurring when responses occurred between 300 and 400 ms (Lam-Cassettari et al., 2021). Bornstein and colleagues found that across 11 countries examined, mothers most frequently respond to their children within two seconds of the end of the child’s utterance regardless of the culture of origin (Bornstein et al., 2015). Further, when experimentally manipulated, infants adapted their babbling to caregiver responses with semantic and temporal contingency and not to those temporally discontinuous with the flow of interaction (Goldstein and Schwade, 2008). Socially contingent interaction hinges upon temporal contiguity; without it, the infant may not take her turn, the toddler may not pair the referent with the symbol, or the preschooler may not link the second step of the direction to the first.

Social contingency, then, is social interaction that respects both semantic contingency and temporal contiguity to sustain the social interaction and, importantly, to increase the odds that provided language will be accurately mapped to referents. Tamis-LeMonda and colleagues’ longitudinal work on maternal responsiveness and child language outcomes has yielded stunning results—nine and 13-month-old children of caregivers in the 90th percentile and above in responsiveness achieved developmental language milestones (e.g., first words, word combinations) four to six months earlier than their age-mates of caregivers in the 10th percentile and below (Tamis-LeMonda et al., 2001; Tamis-LeMonda et al., 1998).

So, over time, what started as objective measures of simple observable behaviors, such as the child’s eye gaze, has expanded to hand-following, symbol-referent pairs, infant-directed speech, and temporally linked behavioral measures all aimed at characterizing what was once simply “joint attention.” The study of joint attention has grown into a veritable subfield of its own, bringing social and cognitive developmental psychology together. Ultimately, it is not merely the presence of social interaction that matters, but the type and quality of social interaction. This subfield has moved on from broad measures of looking patterns to tightly operationalized measures of caregiver, child, and dyadic behavior via moment-to-moment behavior analysis, and, most recently, the burgeoning field of behavioral neural synchrony.

## 4 Neural underpinnings

As the lens sharpens on social cognitive development, socially contingent interactions remain a core driver of later language learning. Decades of behavioral research drove the development of time-locked, moment-to-moment behavioral and physiological measures of synchrony in social relationships during comfortable interactions (Feldman, 2006). Methodological and analytical advances also allowed the use of neurological measures like MRI, fMRI, MEG, fNIRS, and EEG for these purposes (e.g., Redcay and Schilbach, 2019; Paterson et al., 2006; McDonald and Perdue, 2018; Luk et al., 2020; Gaudet et al., 2020). With the advent of synchronous neurological measurement, sometimes referred to as “hyperscanning” (Babiloni and Astolfi, 2014), the field is now prepared to investigate synchronous neurological activity in both social partners associated with the rich social interactions that found ongoing social and cognitive development (e.g., Kinreich et al., 2017; Reindl et al., 2018; Piazza et al., 2020; Nguyen et al., 2023). Importantly, however, these measures are differently attuned and allow us to examine different aspects of the contingent process. Each has its own strengths in terms of temporal resolution, appropriateness for data collection in early childhood, and application to naturalistic social interactions.

### 4.1 Methods for studying baby brains: strengths and weaknesses

Social contingency is a key factor linking social development to language outcomes (Tamis-LeMonda et al., 2014; Hirsh-Pasek et al., 2015; Goldstein and Schwade, 2008). To understand developmental social contingency at a finer grain, researchers must employ neural measures that capture the timing of input and output during activities or interactions reliably with infants, toddlers, and preschool-aged children that mirror real social interactions as much as possible. The neurological measure must allow for temporal specificity while managing the measurement of young children who are likely to move, may exhibit difficulty complying with directions, and may not tolerate the sensory experience of wearing or sitting in enclosed or noisy neurological equipment. To measure moment-to-moment brain activity during *social* interactions, the methodology must also allow for either naturally occurring social interaction or a carefully designed paradigm aimed at measuring specific features of those interactions under strict control.

#### 4.1.1 Timing

Social contingency is defined by its semantic and temporal contingency (Tamis-LeMonda et al., 2014). Essential to the purpose of neurological measurement of high-quality social interactions, then, is the measurement of brain activity at the moment-to-moment level to better understand the characteristics of these interactions driving the relation between social contingency and language learning (Marriott Haresign et al., 2022; Marriott Haresign et al., 2024). The efficacy of these measures in capturing activities that represent real-world social engagement, however, hinges on the age and compliance of the participant. Some measures, like magnetic resonance imaging (MRI), are employed for the purposes of monitoring structural changes to brain regions, while functional measures like electroencephalography (EEG), magnetoencephalography (MEG), functional MRI (fMRI), and functional near-infrared spectroscopy (fNIRS) are designed to capture brain activity in real-time during activities of interest.

EEG and MEG are both direct physiological measures of brain activity. EEG measures electrophysiology of nearby cortical regions via scalp electrodes and MEG measures the very small magnetic fields generated during neuron firing via superconducting quantum interference devices (SQUIDs) held in a large helmet under which the participant sits (Kao and Zhang, 2019). As direct measures of the electrical current and magnetic activity characteristic of neuronal firing, EEG and MEG maintain the unique ability to measure brain activity with tight temporal specificity (Turk et al., 2022a; Endevelt-Shapira and Feldman, 2023; Gaudet et al., 2020; Lew et al., 2013). While the signals are direct indicators of brain activity, one limitation of EEG scalp electrodes is spatial specificity. Neuronal signaling in cortical regions becomes diffuse after passing through several layers of cerebrospinal fluid and cortical, dural, and bone tissue before reaching the scalp (Chen et al., 2019). Thus, while the signal is conducted quickly and can be measured precisely, technological and analytical advances are needed to improve the spatial resolution of EEG (Chen et al., 2019). Alternatively, spatial resolution for MEG is not disrupted by the distance between the region of interest and the SQUID. The magnetic field is not disrupted by the tissue through which it passes, thus allowing better spatial resolution and data on deeper brain regions (Hämäläinen et al., 1993; Chen et al., 2019).

fMRI and fNIRS are both indirect measures of brain activity. They measure blood flow in the brain via radioactive tracer and blood-oxygen level dependent (BOLD) magnetic imaging, and yield superior spatial resolution compared with EEG and MEG (Graham et al., 2015; Aslin et al., 2015; McDonald and Perdue, 2018; Wilcox and Biondi, 2015). They are considered functional measures of brain activity, capturing the pathways and communications among brain areas during a specific activity. As indirect measures, fMRI and fNIRS rely on established norms and averages to describe the expected lag between actual brain activity and blood perfusion measured via BOLD (Alonso et al., 2024; Su et al., 2023; Bazán and Amaro, 2022). Thus, while this lag can be estimated at the level of seconds, direct measures like EEG have millisecond level temporal specificity (Bazán and Amaro, 2022). As technology improves, researchers’ inferencing continues to improve regarding this temporal linkage, but as an indirect measure of brain activity, there may always be temporal limitations inherent in these functional BOLD-driven measures.

So, among the available neuroimaging technologies, research questions and subsequent methodologies will dictate the appropriate measure. In the case of social contingency, we argue, it is essential to privilege temporal resolution to allow for careful, time-locked analysis of social interactions and their features below the level of one second.

#### 4.1.2 Children

Central to the investigation of cognitive and social development is measuring brain activity in infants, toddlers, and preschool-aged children. Collecting data directly from young participants undergoing development and participating in real social interactions is a unique challenge considering developmental behavior, compliance, and tolerance for new people, places and experiences.

While institutional review boards require that children assent study procedures as soon as they are able, studying infants and toddlers often means that only caregivers consent to procedures the child may undergo. Recent studies suggest improving children’s understanding of what is going to happen and providing agency in the process improved participation rates (e.g., 72% for 2-3-year-olds; Norton et al., 2022; Adams et al., 2024). When children are provided control over their participation and offered the opportunity to understand what will happen, they are more likely to comply and even participate in a more naturalistic manner. What this means in practice is that even though young children might not be able to consent, they should be provided the same agency and body autonomy we would provide adults.

While providing agency may improve compliance, it is essential to keep in mind that children, even those intending to comply, may be unable to sit for extended periods of time, experience new tactile or auditory stimuli, or engage with unfamiliar adults. fNIRS and EEG carry the benefits of data collection outside of a large magnet or tube but do require the child to wear a cap or net on their head, often tethered with wiring connecting the electrodes or sensors to an amplifier, which then communicates to a larger data collection computer via corded or wireless transmission. Modern technologies allowing for wireless data transmission from a mobile cap and amplifier to the data collection computer bring their own unique challenges regarding noise (e.g., the child can move more freely and is more likely to make physical contact with equipment), transmission (e.g., failing or intermittent Bluetooth data connections), and ecological validity (e.g., the weight or presence of the amplifier, affixed to the bottom of the cap or worn on the back of the child may intervene with natural interaction styles; Troller-Renfree et al., 2021; Noreika et al., 2020). fMRI and MEG require that the child sit or lie in one place in one position for an extended period. All four measures, fNIRS, EEG, fMRI, and MEG, are sensitive to motion artifacts to varying degrees, though new mobile systems for fNIRS and EEG have improved data collection under these conditions and allow the child to sit in a more comfortable or familiar seat, or even walk short distances from the data collection computer (e.g., Troller-Renfree et al., 2021; Throm et al., 2023). Additionally, computational techniques aimed at filtering this type of noise in the data are continually under development (e.g., Monachino et al., 2022; Gabard-Durnam et al., 2018).

Methodological approaches including selection of equipment, data cleaning and analytic techniques, and developmental appropriateness of presented tasks are essential considerations when collecting data from young children with their own ideas, emotions, and physical bodies. Neurological methodologies employed with young children must respect the child’s autonomy as it is the researchers’ responsibility to protect participants’ well-being. Additionally, methodological approaches enacted without the comfort or trust of the participant may yield unpredictable data. That is, activity and arousal levels in young children invariably affect equipment reliability and the child’s brain activity itself (Troller-Renfree et al., 2021; Noreika et al., 2020). To control for as many variables as possible, the research environment should be safe, predictable, and developmentally appropriate.

#### 4.1.3 Social validity

Methodological design must account for the validity of the measures conducted. In the case of social contingency, the aim is to measure brain activity associated with naturalistic social interactions. For school-aged children, adolescents, and adults, sophisticated paradigms allow for measurement of real-time brain activity during various activities while sitting still under a MEG helmet or in an MRI scanner (Tsoi et al., 2022; van Atteveldt et al., 2018; Kinreich et al., 2017). These methods often employ familiar television, games, and other activities intended to trigger brain activity similar to the activity of interest. Outside of a scanner tube or helmet, neurological measures allowing the child to move his head more freely improve the likelihood that the signal detected is related to social interaction in the real world.

In the case of social contingency in early childhood specifically, EEG and fNIRS are more mobile options that tolerate some motion in the child and do not require lying in an MRI scanner or sitting under a MEG apparatus. In fact, once the child is capped, she often forgets she is wearing the cap, returning to typical behavior within a few minutes. Study design, then, can more closely align with real-world social scenarios including the use of familiar social partners, measurement during a variety of activities (e.g., Norton et al., 2022; Nguyen et al., 2020b; Quiñones-Camacho et al., 2020; Liao et al., 2015), and even measurement outside of the controlled laboratory space (Troller-Renfree et al., 2021; Dikker et al., 2017; Dikker et al., 2021; Bevilacqua et al., 2019).

So, limitations remain for all available neurological measures for this application. *All* presented measures are sensitive to artifacts and noise introduced by the environment including motion. Thus, employing any method during naturalistic interactions with infants and young children poses significant methodological and analytical challenges. Further, fMRI and MEG require the child to be locked into a physical space that prevents natural responses to stimuli related to these research questions. Clever design and technological advances will likely continue to manage and reduce this limitation; for example, studies have used cameras, mirrors, and proximity to attenuate these limitations (e.g., Hirata et al., 2014; Hasegawa et al., 2021). Labs have also begun to adapt systems for upright-sitting MEG data collection (e.g., Bosseler et al., 2024) and even for a smaller MEG wearable device using optically pumped magnetometers (e.g., Holmes et al., 2023). Not all technologies have been trialed with child participants (e.g., Holmes et al., 2023). For now, limited data are available. EEG and MEG appear to have the temporal specificity edge, though as equipment technology and computational approaches continue to improve, this may not remain the case. Young children may display difficulty with tolerating loud sounds, as are present for fMRI and MRI imaging, sitting still, as for MEG and f/MRI studies, or for wearing an EEG or fNIRS cap or amplifier. The EEG or fNIRS alternatives seem the least invasive and troubling to children, though research design, establishing rapport, and offering agency wherever possible are important factors in gleaning usable, meaningful neurological data, no matter the modality. Finally, at present, EEG and fNIRS remain the most mobile neurological technology, with fNIRS particularly resistant to motion artifacts (e.g., Bulgarelli et al., 2023; Piper et al., 2014). This mobility should increase the ecological validity of the social interactions measured.

What Adamson and Bakeman illuminated is that the key to understanding language development lies in dyadic interaction. Even with the weaknesses of the extant measures, the field is moving toward the ability to capture brain activity in a young child undergoing social development. Thus, from these neurological measures, the field has pushed forward into dyadic measures of neurological activity via neural synchrony, or hyperscanning. These advances allow researchers to examine the *dyadic* qualities Adamson and Bakeman argue are central to socially contingent interaction and subsequent language development.

### 4.2 Neural synchrony

Layering onto the joint attention and engagement skills described by Adamson and the salient qualities of contingent interaction described by Tamis-LeMonda, Ruth Feldman moved the field from behavioral, to physiological measures of synchronous interaction such as heart rhythms, to neurobiological measures like oxytocin, and to neurological synchrony (Feldman, 2006; Feldman et al., 2011; Feldman et al., 2013; Feldman, 2016). Neural synchrony work was initially confined to adult pairs (e.g., Kinreich et al., 2017), though improved technology and methodologies have since allowed for investigations including even young children. This neural synchrony work has been conducted in infant-caregiver pairs using EEG, MEG and fNIRS (e.g., Leong et al., 2017; Piazza et al., 2020; Levy et al., 2017; Levy et al., 2021; Lin et al., 2023; Wass et al., 2018). Only recently has neural synchrony during naturalistic social interactions between caregivers and children become feasible. fNIRS and EEG technology allow for the measurement of cortical activity during social interaction without being fixed to one physical space (i.e., a scanner). Limitations to these measures related to artifacts and noise introduced by the environment, motion, and difficulty engaging infants in interactions with electrodes or sensors on their heads previously limited the viability of these more naturalistic methods. Methodologists and analysts continue to grapple with these challenges in measuring naturalistic interactions.

In the meantime, research demonstrated patterns of connectivity and structure in early development relating to language, employing MRI methods recording from individual participants (e.g., Merz et al., 2020; Romeo et al., 2018b; Romeo et al., 2018a; Paterson et al., 2006). In two hallmark studies, Romeo uncovered the structural and connective relations among well-studied language areas, Wernicke’s (left posterior superior temporal) and Broca’s (left inferior frontal) areas. Using structural MRI, Romeo examined the microstructure of the white matter tract linking these two areas, the superior longitudinal fasciculus (SLF). These data suggested a relation between adult-child conversational turns, measured via automated Language Environmental Analysis (LENA) during naturalistic interactions and connectivity in the SLF, such that increased conversational turns trended with increased evidence of connectivity, independent of socioeconomic status (SES) and language exposure quantity (Romeo et al., 2018b). In a functional MRI study, Romeo also identified increased activation *during* a story-listening activity in four-to six-year-olds. Children exposed to more adult-child conversational turns evidenced greater Broca’s area activation (Romeo et al., 2018a). Relatedly, Merz and team recorded greater surface area of the left perisylvian region in children experiencing more adult-child conversational turns in the home, also via LENA recording (Merz et al., 2020). Collectively, these structural data reflect increased cortical volume and connectivity in left-dominant language areas for children experiencing more conversational turns. Functional data show that activation patterns reflect the underlying structural and connective evidence.

The findings are compelling but require the child to remain inside a scanner rather than interacting naturally with a communication partner. Thus, this research area has pushed into real-time synchronous recording during more naturalistic interactions to allow for a better understanding of the moment-to-moment co-activation of infant and caregivers’ brains during contingent social interaction (e.g., Norton et al., 2022; Nguyen et al., 2020a; Nguyen et al., 2020b; Turk et al., 2022a; Turk et al., 2022b).

Early studies employing hyperscanning methods with caregiver-infant dyads focused first on associating both participants’ brain signals via fNIRS with established behavioral measures such as mutual gaze, joint attention, and child-directed speech (e.g., Piazza et al., 2020). For example, Piazza and colleagues identified neural synchrony only during joint interaction phases during naturalistic interactions recorded in the laboratory setting. Prefrontal cortex activity in the infant and adult social partner was linked during mutual gaze and infant emotionality (e.g., smiles). Overall, the authors also reported that infant brain dynamics appeared to precede or even lead the adult brain activity (Piazza et al., 2020). In another fNIRS study, this time later in early childhood with children aged 3.5 to 4.5, Piazza and colleagues identified synchronized parietal activity during a focused book-reading task intended to introduce novel vocabulary. This joint learning opportunity was reflected in parietal activity in both participants and the neurological activity was associated with successful learning of the novel words presented in the book (Piazza et al., 2021). Additional fNIRS studies by Nguyen and colleagues link conversational features, neural synchrony, and language development (e.g., Nguyen et al., 2020b; Nguyen et al., 2023). The authors found that even when infants were just four to six months, dyads with higher bidirectional turn-taking also exhibited higher neural synchrony and that the turn-taking behavior was associated with later expressive vocabulary (Nguyen et al., 2023).

Hyperscanning studies employing EEG have yielded similar findings linking individual attention, joint attention, and conversational features. In one study, shared eye gaze was associated with synchronous brain activity between caregiver and infant. The infants vocalized more during this direct, shared gaze during live social interactions and, when vocalizing, neural synchrony was enhanced (Leong et al., 2017). During a structured EEG hyperscanning study, Bánki and colleagues showed that when the presentation of visual stimuli was paired with naturalistic communicative cues (verbal and gestural), joint attention to the presented images increased and child visual processing increased, as measured by steady-state visual evoked potentials in mother and infant (Bánki et al., 2024). Endevelt-Shapira and Feldman collected dual EEG data during face-to-face interactions that show that mother-infant neural synchrony was positively associated with maternal responsivity and negatively associated with maternal intrusiveness (Endevelt-Shapira and Feldman, 2023). So, dual EEG has allowed researchers to show that, during more naturalistic interactions including face-to-face social interaction paradigms, characteristics of behavioral synchrony relate to neural synchrony. Importantly, however, the behavioral and neural markers of synchrony do not perfectly align. That is, the temporal specificity of neural synchrony data allows for a truly dyadic feature of interaction to emerge, where behavioral coding of social interactions appears, by comparison, a blunt measure of these rich interactions. Of note, however, is the inherent difficulty in capturing the naturalistic social environment of an infant or toddler. The above paradigms rely on children sitting still and engaging in paradigms aimed at mirroring naturalistic interactions, rather than measuring true social interaction as it would exist for infants and toddlers in their own environment.

Several recent publications highlight the feasibility of EEG hyperscanning or single-recordings during dyadic interactions in early childhood and in more naturalistic settings, such as engaging with age-appropriate toys at a table in the lab or even in their own home (e.g., Norton et al., 2022; Turk et al., 2022a; Troller-Renfree et al., 2021). It is essential to note, however, the limitations of neurological measurement with respect to simultaneously managing signal quality and the ecological validity of the naturalistic social interaction setting of an infant or toddler. As the technology advances to meet the needs of the science, researchers are beginning to collect objective brain data demonstrating the value of social connection for word learning. Piazza and colleagues, for example, employed a digital storybook paradigm with preschoolers (Piazza et al., 2021). Though the materials were experimentally manipulated and the child seated in a chair in the lab, reading a digital storybook with a social partner matches the naturalistic social environment. Research also supports the timing of these social interactions for optimal learning (e.g., Lam-Cassettari et al., 2021; Leong et al., 2017).

Advances in neurological measurement of social interaction have allowed researchers a window into moment-to-moment brain activity during social contingency with respect to semantic contingency and temporal contiguity. In a novel MEG setup, for example, the I-LABS team at the University of Washington recorded just the five-month-old infant’s brain activity during live, face-to-face interaction (Bosseler et al., 2024). The MEG apparatus was oriented to allow the child to sit upright while the caregiver interacted either directly with the child or with an adult just out of the child’s visual field. Previous MEG studies were limited in replicating a naturalistic environment or interaction due to the positioning of the equipment. In fact, just three of the total sample of 41 infant participants did not tolerate sitting under the MEG equipment for this experiment. With these new methods, Bosseler and colleagues tested the effect of naturalistic social and nonsocial stimuli on the infant brain *and* measured language outcomes longitudinally. Activation in attention and sensorimotor regions at five-months of age was associated with vocabulary growth from 18-to 30-months and with 24-month expressive vocabulary (Bosseler et al., 2024). The I-LABS team also conducted the first MEG hyperscanning studies, including a caregiver-child interaction with preschoolers showing that naturalistic social interaction consisting of turn-taking yielded enhanced brain synchrony in parietal and frontal regions (Lin et al., 2023).

Collectively, these neural data reveal that socially contingent interactions are not just a behavioral phenomenon, but a lever in the brain development machinery. Brain structure (e.g., Romeo et al., 2018b), connectivity (e.g., Romeo et al., 2018a), and functional activation (e.g., Leong et al., 2017; Piazza et al., 2021) seem to reflect that exposure to reciprocal interaction *builds* brain structure and connectivity during these rich interactive moments. These connectivity and activation patterns bear out in the child’s social engagement, communication skills via scaffolded statistical learning, and, it seems, perhaps much more. The case thus far has been limited to a robust behavioral literature on social growth and language development through contingent interactions. But recent work suggests that language might be only the beginning of the profound relations between social development and outcomes hinted at by Kuhl and her team (Kuhl, 2007; Meltzoff et al., 2009). Behavioral and neural synchrony research also links social contingency to executive function skills like attention and emotion regulation, and additional cognitive skills like problem solving.

## 5 Moving beyond language to neural synchrony in other developmental outcomes

As technology advances from basic science research design to contextualized measurement of real-world development, the field is asking bigger questions about the role these social interactions might have for developmental outcomes from language to beyond. As these rich, socially contingent interactions are revealed, familiar social and vocational outcomes come to light (e.g., social responsiveness—Lasch et al., 2023; attachment—Ainsworth, 1969; social and behavioral competence at school age—Vaughan Van Hecke et al., 2007; Sheinkopf et al., 2004).

What happens if we broaden the lens to investigate the role of measured social interaction quality in the development of other cognitive skills like executive function including attention? Rolling back the footage to maternal responsivity and the work of Tomasello, Goldstein, and Tamis-LeMonda, perhaps the dyadic interaction quality revealed in early social contingency measures is actually *creating* the neurological fabric leading to cognitive outcomes going beyond language.

### 5.1 Attention

Research on attention even in early infancy revolves around the development of the sensory system. That is, attention for the purpose of engagement with the world. One key sense through which humans engage is vision; and vision has received most of the work in this research field. From this visual attention early in infancy develops an endogenous or volitional use of visual attention, allowing the infant to orient to stimuli of interest. Around six months, infants begin to follow another’s gaze (Morales et al., 1998) and around 12 months, to direct the attention of an interaction partner. Sustained attention, attention shifting, and sustained interactions continue to improve over the first years of life (Bahrick et al., 2018). As infant attention develops from an exogenous or externally driven sensory skill to an endogenous cognitive skill motivating early social interactions, attention grows from the product of a functioning visual system to an intricate, volitional, social skill. It is here that attention becomes critical in the social interactions that predict language skills (Masek et al., 2021).

Social contingency, Masek argues, is founded upon the sustained attention skills that Adamson described as pre-requisite to joint attention: the triadic, shared interaction experience key to early social experience and learning (Masek et al., 2021). In fact, there is evidence that infants with higher caregiver-reported attention skills also had stronger language skills (Dixon and Shore, 1997; Dixon and Smith, 2000). Then, as attention develops, from following attention to directing others’ attention to joint attention, child development sees an explosion of social skills with or without language as language growth, too, explodes. That is, these skills are interdependent and non-linear.

EEG studies have shown that attention and learning in infants during social interactions relate to both the focus of their social partner’s attention and to neural responsivity (e.g., Wass et al., 2018; Phillips et al., 2023). Fine-grained time series analyses facilitated by use of EEG paired with physiological data and painstaking behavioral coding allows for further investigation of key interactions. Wass and colleagues have developed fine-grained time series analyses employing Granger causality to dig into the neurological, physiological, and behavioral dyadic qualities of social interaction between caregiver and infant. In one such hyperscanning study, Wass showed that caregivers’ neural activity was greater when their infant was attending to an object during social play (as compared to solo play) *and* that infant attention increased with this caregiver neural response. This detailed time-series analysis is unique to this methodology and analytic technique, allowing the researchers to test the temporal sequence of the interacting neural and behavioral data. Analyses conclusively determined, for example, that caregiver EEG activity *followed* infant attention, but not vice versa. That is, the adult tracked the infant’s attention and when this occurred, infant attention increased (Wass et al., 2018). The neural measure seems to reflect a relation between mutual awareness of a social partner’s attention and the child’s actual focus of the attention. EEG collected from infants as young as 10 months reveals that early in development children learn to expect social contingency from their social partners. In the moments following mutual attention led by the infant, infant alpha was suppressed, indicating the infant expected the social partner to share their attention (Phillips et al., 2023). These researchers also reported increased sustained attention when it was mutual as opposed to independent (Phillips et al., 2023).

Socially contingent interaction, then, is not operating on the singular level of the child or the adult, but at a dyadic level. It is also not operating on a singular level of basic visual attention to an object, but rather multiple levels including visual attention broadly defined, caregiver *and* child metacognition around the social pair’s focus of attention, and the statistical learning for the child brought about by this basic attention paired with social metacognition. With a caregiver sensitive to the child’s focus of attention, developmental level, support needs, and learning interests, any environment becomes a learning environment for the child as experiences become opportunities to make meaning around symbolic referents (i.e., word learning), learn about solving problems, regulate one’s emotions, and sustain attention. This learning is also not linear or unidirectional—as the child learns, the adult unconsciously catalogs the child’s status in the socially contingent interaction. As the pair attune to one another, they optimize their attention in the moment, but *also* for future interactions. As the research continues to build in this area, it looks more and more as if social interaction and the intertwining of child and adult social contingency might lie at the foundation of attention.

### 5.2 Executive functioning, emotion regulation, and problem solving

Attention is related to wider discussions of additional cognitive and emotional skills such as executive function, including inhibition, cognitive flexibility, and working memory (Zelazo and Carlson, 2020), emotion regulation (Masek et al., 2021), and problem solving (Nguyen et al., 2020b; Nguyen et al., 2021). It is becoming clear that early attention and social interaction skills as noted above, beget later cognitive and emotional outcomes including skills like inhibition, more sophisticated attention skills, and emotion regulation (Masek et al., 2021; Masek et al., 2023; Masek et al., 2024). As Carlson notes, these developmental skills are “meaningful, measurable, and malleable” (Carlson, 2023) and relate to numerous key developmental outcomes. For example, emotion regulation is linked to social development including social metacognition like theory of mind (Zelazo and Carlson, 2020). Cognitive and emotional executive function skills predict school readiness for math and reading directly and indirectly via goal-directed problem-solving, self-regulation, and flexible adaptation (Zelazo and Carlson, 2020; Goldin-Meadow et al., 2014), including later language comprehension and literacy (Bleses et al., 2016). Armed with the ability to attend, interact, and engage, and an interaction partner attuned to the temporal and semantic cues of the developmental environment, the infant is primed for cognitive development via scaffolded statistical learning.

Executive function, initially eschewed as an outcome for joint attention studies because of its dubious measurement before preschool age, is now linked by longitudinal evidence showing a connection between joint attention early in development and cognitive and emotional executive functions later in development (e.g., Miller and Marcovitch, 2015; Brooks and Meltzoff, 2008). Work investigating executive function prior to three years of age yielded inconsistent or null results, likely due to immature executive functioning (Miller and Marcovitch, 2015; Lasch et al., 2023). At three years and beyond, however, skills like delayed gratification (e.g., Miller and Marcovitch, 2015; Vaughan Van Hecke et al., 2012), self-regulation (e.g., Feldman et al., 2006; Gago Galvagno et al., 2019), and theory of mind (e.g., Brooks and Meltzoff, 2008) appear tied to established social relationships. In fact, Mundy argues that the “integration of early executive, motivation, and social-cognitive processes suggests that infant joint attention may be fundamental to the development of social competence in childhood” (Mundy and Sigman, 2015).

Neural synchrony measures now offer a new perspective on the relations between this broader suite of executive function skills and joint attention. For example, a recent fNIRS hyperscanning study on delayed gratification linked neural synchrony and executive function skills. Zhao and colleagues paired three-year-olds with their caregivers and with strangers during a sticker activity. The adult was directed whether to model selecting fewer stickers and receiving the reward immediately or selecting more stickers that required placing them in a bucket for later retrieval. Children learned delaying gratification by selecting more stickers to receive later when paired with their caregiver. Furthermore, the fNIRS-recorded synchronous brain activity during the caregiver’s execution of the task, and the child’s observation and inferencing of that execution, predicted the child’s choice to delay gratification. Child brain activity alone was not predictive of delay of gratification (Zhao et al., 2023). Learning delay of gratification by observation is an established theory (Zelazo and Carlson, 2020), but the addition of hyperscanning allowed for further conclusions around the underpinnings of this process that could lead to better understanding of psychopathologies or could be employed in interventions.

Similarly, Reindl and her team identified increased coherence via dual fNIRS recording between five-to nine-year-olds and familiar, as opposed to unfamiliar, social partners during a cooperative task. Further, this coherence was related to child emotion regulation. That is, when cooperatively interacting with a familiar social partner, children’s brains were more in sync with their partners and they were better emotionally regulated than when interacting with a stranger (Reindl et al., 2018). Quiñones-Camacho and her team have also linked dual fNIRS data during social interactions between four-to five-year-olds and their caregivers with internalizing and externalizing behaviors. Higher neural synchrony during play was associated with a faster decline in internalizing behaviors over the following year-and-a-half of development (Quiñones-Camacho et al., 2022). This downward trajectory is expected and typically considered an analog to increasing emotional regulation (Quiñones-Camacho et al., 2022). Thus, it seems that socially contingent interaction may found neural connections that serve child development beyond just social relationships and language development, but perhaps also executive function and a broader cognition.

Recent advances in neural synchrony measures allow for investigation into the relation between interaction quality and problem solving. For example, in one study with preschoolers and their mothers, children solved tangram puzzles. fNIRS data reflect increased task performance when the child cooperated with their mother and neural synchrony was present (Nguyen et al., 2020b). Neural synchrony, the authors argue, serves as a biomarker for rich interaction quality. Thus, stronger interaction quality was associated with improved cooperative problem solving. Similar results were found in adult pairs and school-aged children paired with their mothers (Fishburn et al., 2018; Reindl et al., 2022). Combining present evidence on later development and into adulthood supporting neural synchrony’s benefits to problem solving, Nguyen’s work shows the value of exploring the development of these problem-solving skills from a neural synchrony perspective, earlier in development. Research on the way in which behavioral neural synchrony might foster problem solving in younger children is fertile ground ready to be further explored.

## 6 Neural data advance the literature beyond the behavioral data

A question often posed is whether knowledge of neural data—here neural synchrony—advances our understanding of outcomes beyond those already known through behavioral measures. As Weisberg and her colleagues noted, neuroscientific data create more interest than do behavioral data, even when it does not add new information to the argument (Weisberg et al., 2008). Thus, it is essential that researchers creatively employ neurological measures paired with behavioral measures to enrich the understanding of developmental phenomena.

In the case of neural synchrony data, there are four ways in which the research adds immeasurably to the science of how social interaction might impact cognitive growth. The first is by examining contingent interactions in units smaller than one second. Social interaction has proved difficult to fully measure behaviorally because the dance of the interaction occurs at a micro-interaction level. Furthermore, timing matters because caregivers who adapt to the flow of the back-and-forth interaction in the moment, are generally more sensitive and responsive parents. As addressed above, sensitive and responsive parenting yields better developmental outcomes (e.g., Tamis-LeMonda et al., 2001; Tamis-LeMonda et al., 2014; Landry et al., 2008).

Second, more precise indicators of timing matter because they allow researchers to see which behaviors come online or go offline amidst the interaction. When in a socially contingent stream is attention activated? How is it modulated across time within socially prominent or less prominent moments? That is, by looking at dyadic changes and adaptations along with brain activation, we can witness the unfolding of development over time.

The third key contribution of the neural data is that it offers a better measure of dyadic rather than singular data. That is, most of the data in the field talk about the need to study dyads, but then resort to asking what the adult is doing at point X and later focusing on what the child is doing at point X. Neural synchrony enables a clear look at dyadic interactions *in situ*.

Fourth and finally, researchers generally select one area of interest—be it attention or social activation to study in the course of an experiment. Surely, these behaviors are more interdependent than they are independent. While it is convenient to isolate a construct behaviorally, it is essential to find new methods that clearly articulate the interrelations between these seemingly separate behavioral constructs. Neural synchrony studies allow us to peek at these interdependencies in the moment and over time.

For all of these reasons, the study of how social brains might support cognitive development is enriched by going beyond the constraints of behavioral data to add neural data. These data surely hold some surprises as we unpack the timing of the interdependencies in the flow of everyday interactions.

## 7 Frontiers

While some literatures are more established than others, key findings exist linking social interaction to cognitive outcomes. These connections are well-established for language, as a natural core feature of social interaction and cognitive development, though a strong case is also made for other cognitive processes including attention, executive function, and problem solving. With scaffolded support from adults, children attend for longer and make symbol-referent pairs that drive not only language learning but problem-solving, self-regulation, and perhaps more. These social-cognitive processes are tightly linked developmentally and require precise tools to better untangle their relations.

As equipment and analytic technologies continue to advance and more data become available, behavioral neural synchrony offers a new window into development that will allow moment-to-moment analysis of behavioral, physiological, and neurological data. Researchers must continue to drill down to real brain-to-brain relations, reducing noise via methodological and analytical controls limiting behavioral artifacts that may amplify or reduce synchrony. Linking well-established behavioral and single brain recording neural literatures alone will not be sufficient. Instead, dual-recording neuroscience capitalizes on these vast literatures in a way that allows the aperture to focus even more tightly on the temporal linkages present in dyadic interaction in the moment. Rather than describing the behavior of one individual or documenting the neural activity of one individual, neural synchrony provides access to new frontiers allowing a finer grained analysis of the social and cognitive interdependencies borne out of social interaction early in development.

## 8 Conclusion

Budding evidence that other areas of development may be impacted, before infants even use symbolic language, suggests that perhaps socially contingent interactions shape more than just language development. Indeed, perhaps the social gating system provides the foundation for a broader cognition—from attention to executive function, to problem solving.

Evidence for statistical learning early in development is not restricted to language learning (Saffran et al., 1996; Aslin, 2017). Thus, the scaffolding provided during socially contingent interactions, providing the child with a sharper lens through which to view the world, can apply to additional cognitive skills. That is, with a supportive social partner, the child can sustain attention longer, participate in more social or conversational turns, develop symbolic communication, auditory comprehension, and a slew of cognitive and developmental skills farther down the cascade. Development, after all, is rarely about the growth or change of one isolated skill, but, as neuroscientists describe, the proliferation and pruning of synaptic connections across the brain. The development of symbolic communication is one piece of the evidence puzzle supporting the foundational social relationship that drives the mapping of cognition in the brain.

As behavioral neural synchrony research continues, evidence grows in favor of Kuhl’s hypothesis (Kuhl, 2007). Socially contingent interactions drive neurological activation as the scaffolding of the developmental environment. Thus, early relationships are central to child development. With decades of behavioral and neural data to bolster the field’s research questions, neural synchrony measures are poised to offer a deeper understanding of the role of social connection in cognitive development via moment-to-moment *dyadic* analysis.

Neural synchrony research methods now fuel the flames at the base of cognitive development research. With these data, the flames continue to grow as the field moves toward a better understanding of the role of early social interaction as the rocket readies to launch from behavioral synchrony to language, and beyond. The view of social cognitive development from space is vast, with endless possibilities for inquiry remaining.
